# Structures of Eukaryotic Ribosomal Stalk Proteins and Its Complex with Trichosanthin, and Their Implications in Recruiting Ribosome-Inactivating Proteins to the Ribosomes

**DOI:** 10.3390/toxins7030638

**Published:** 2015-02-25

**Authors:** Andrew K. H. Choi, Eddie C. K. Wong, Ka-Ming Lee, Kam-Bo Wong

**Affiliations:** School of Life Sciences, the Chinese University of Hong Kong, Shatin, Hong Kong, China; E-Mails: s1155004528@cuhk.edu.hk (A.K.H.C.); s1155029371@cuhk.edu.hk (E.C.K.W.); leekaming@cuhk.edu.hk (K.-M.L.)

**Keywords:** ribosome, trichosanthin, ricin, stalk, ribosome inactivating proteins, elongation factors

## Abstract

Ribosome-inactivating proteins (RIP) are RNA *N*-glycosidases that inactivate ribosomes by specifically depurinating a conserved adenine residue at the α-sarcin/ricin loop of 28S rRNA. Recent studies have pointed to the involvement of the *C*-terminal domain of the eukaryotic stalk proteins in facilitating the toxic action of RIPs. This review highlights how structural studies of eukaryotic stalk proteins provide insights into the recruitment of RIPs to the ribosomes. Since the *C*-terminal domain of eukaryotic stalk proteins is involved in specific recognition of elongation factors and some eukaryote-specific RIPs (e.g., trichosanthin and ricin), we postulate that these RIPs may have evolved to hijack the translation-factor-recruiting function of ribosomal stalk in reaching their target site of rRNA.

## 1. Introduction

Ribosome inactivating protein (RIP) belongs to a family of proteins that inactivate ribosomes. Most RIPs discovered were isolated from plants (e.g., ricin, trichosanthin, maize RIP, pokeweed antiviral proteins), but a few RIPs were found in bacteria (e.g., Shiga and shiga-like toxins) [1]. All RIPs contain an RNA *N*-glycosidase catalytic domain that enzymatically removes a specific adenine base from the 28S rRNA. In a classical study by Endo and co-workers, the *N*-glycosidase domain of RIP (e.g., ricin A-chain) can depurinate the base of A4324 of rat 28S rRNA and thus inactivates the ribosomes [2]. A4324 is located next to the cleavage site of α-sarcin (G4325–A4326). As a result, this segment of 28S rRNA is later called the α-sarcin/ricin loop (SRL) [3]. It has long been established that modification by ribotoxin on the SRL reduced the ability of ribosomes to bind elongation factors and activate their GTPase activity [4]. Recent structural studies of ribosomes in complex with elongation factors showed that the SRL is located next to the GTP binding site and could play direct role in activation of GTP hydrolysis [5]. It is anticipated that depurination at A4324 should change the conformation of the SRL, resulting in the inability of the ribosomes to activate GTP hydrolysis that is needed to drive protein translation.

RIP can depurinate naked rRNA, but the activity was more than 80,000 fold slower when compared with the whole ribosome [6]. Moreover, some RIPs, like ricin and trichosanthin, can only inactivate eukaryotic ribosomes but not bacterial ribosomes, while the structure of SRL is highly conserved among all bacterial and eukaryotic ribosomes. These observations strongly suggest that ribosomal proteins must play a role in facilitating the action of RIPs. The SRL together with the lateral stalk of ribosomes constitute part of the GTPase activation center [7], which is involved in binding of elongation factors and activation of GTP hydrolysis [8,9,10]. This review focuses on how the structures of eukaryotic stalk provide insights into the recruitment of RIPs to the ribosomes. 

## 2. Bacterial, Archaeal and Eukaryotic Ribosomal Stalks are Different

**Figure 1:**
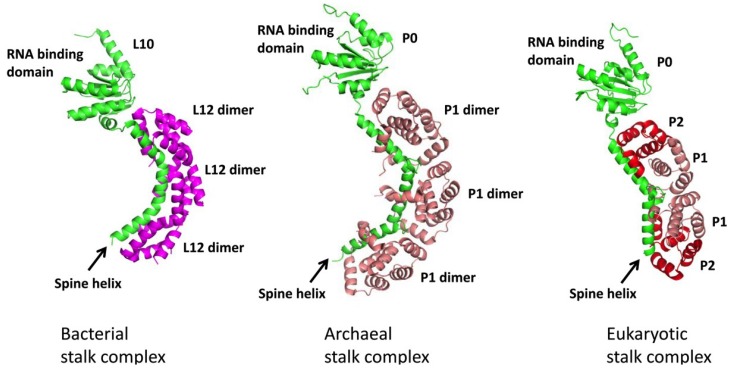
Structural organization of bacterial, archaeal and eukaryotic ribosomes. Structures of bacterial, archaeal stalk complex were determined by X-ray crystallography [11,17], while the structural model of eukaryotic stalk complex was predicted as described [27]. Bacterial stalk complex is consisted of L10 (green) and 2 to 3 copies of L12 dimers (magenta), while archaeal stalk complex is consisted of P0 (green) and 3 copies of P1 homodimers (salmon). On the other hand, eukaryotic stalk is consisted of P0 (green), P1 (salmon) and P2 (red) in 1:2:2 stoichiometry. Two copies of P1/P2 heterodimers bind to the spine-helices of P0, presumably adopting a P2/P1:P1/P2 topology [19,24].

The lateral stalk complex of bacterial ribosomes is formed by L10 in complex with two or three copies of L12 homodimers (Figure 1) [11,12]. Crystal structure of L10/L12 complex from *Thermotoga maritima* was determined by Wahl and co-workers [11]. The L10 protein consists of an *N*-terminal RNA binding domain for anchoring the stalk to the rRNA, and a long spine-helix at the *C*-terminus responsible for binding L12 dimers. The spine-helix of *T. maritima* L10 is divided into three 10-residue segments, each segment binds to the *N*-terminal domain (NTD) of L12. The *C*-terminal domain (CTD) of L12, which is involved in factor binding, is linked to the NTD via a flexible hinge region [13,14,15].

Archaeal stalk complex is formed by ribosomal protein P0 in complex with three P1 homodimers (Figure 1) [16]. The crystal structure of P0/P1 complex from *Pyrococcus horikoshii* was determined by Naganuma and co-workers [17]. The RNA binding domain of P0, except the additionally inserted extended domain (domain II), is homologous to bacterial L10 [18]. Archaeal P0 has three spine-helices connecting to the RNA binding domain and each binds one copy of P1 dimer [17].

The eukaryotic stalk is composed of a pentameric P-complex, with P0 and two copies of P1/P2 heterodimers (Figure 1) [19,20]. The sequence and structure of eukaryotic P0 is homologous to archaeal P0 [21,22,23]. In contrast to archaeal P1 forming a homodimer, eukaryotic P1 and P2 forms heterodimer spontaneously [19]. Eukaryotic P0 is predicted to have two spine-helices, each binds one copies of P1/P2 heterodimers [24,25,26]. Both P1 and P2 have an *N*-terminal domain responsible for dimerization and for binding the spine-helix of P0. The solution structures of the NTD of human P2 homodimer and P1/P2 heterodimer were determined by us recently [19,24]. Although archaeal P1, eukaryotic P1 and P2 share only a low degree of sequence similarity, their tertiary structures are in fact homologous to each other [17,19,24]. On the other hand, P1 and P2 are structurally distinct from bacterial L12. The NTD of P1 and P2 consists of four helices, with helix-1, 2 and 4 forming the dimerization interface. P1 contains an extra turn in helix-1 that forms extra stabilizing hydrophobic interactions with P2 residues, making the formation of P1/P2 heterodimers more favorable than P2 homodimers [24].

The organization of the eukaryotic stalk is perhaps the most complicated due to the diversity of stalk proteins and the formation of heterodimers. For example, yeast has two different copies of P1 (P1A and P1B) and P2 (P2A and P2B) [28], in which P1A forms dimer with P2B and P1B forms dimer with P2A [29]. Unlike the symmetrical archaeal P1 homodimer, binding of two copies asymmetric P1/P2 heterodimers on P0 could result in four possible topological arrangements [19]. Based on structure of the NTD of P1/P2 heterodimer and mutagenesis studies, the topology of P2/P1:P1/P2 is proposed [19,24]. In this model, the conserved residues of helix-3 of two adjacent P1 proteins can form a hydrophobic cavity to accommodate the Tyr-Pro motif on the loop between the two spine-helices of P0.

## 3. Ribosomal Stalk is Involved in Domain-Specific Recognition of Elongation Factors

It has been well established that bacterial and eukaryotic ribosomes use different sets of elongation factors in protein synthesis. For example, bacterial ribosomes use EF-Tu and EF-G but not eEF1α and eEF2. Using a hybrid-ribosome approach, Uchiumi and his co-workers demonstrated that the ribosomal stalk plays a key role in domain-specific recognition of elongation factors. The *E. coli* 50S core ribosome lacking the bacterial stalk proteins (L11 and L10/L12 complex) was reconstituted *in vitro* with eukaryotic stalk proteins (eL12 and P0(P1/P2)_2_ complex). The resulting hybrid-ribosome changes its specificity and uses eukaryotic elongation factors instead [30,31].

It has been shown that the CTD of stalk proteins P1/P2 and L12 are responsible for domain-specific binding of elongation factors [8,9]. The structures of the CTD of bacterial and eukaryotic stalk proteins are different. The CTD of L12 adopts a globular structure [14,15] while that of eukaryotic stalk protein P1/P2 is disordered and flexible [27]. This structural difference in the CTD may facilitate the domain-specific recognition of elongation factors. Uchiumi and co-workers have fused the NTD of L10 with CTD of P0, and showed that the resulting chimeric stalk protein was able to bind two copies of P1/P2 heterodimers and enabled the reconstituted hybrid ribosomes to use eukaryotic elongation factors. This observation suggests that the CTD of eukaryotic stalk proteins are crucial for domain-specific recognition of elongation factors [32]. Similarly, it has been shown that the *C*-terminal consensus sequences of archaeal stalk proteins P0 and P1 are crucial for interacting with archaeal elongation factors [10].

**Figure 2:**
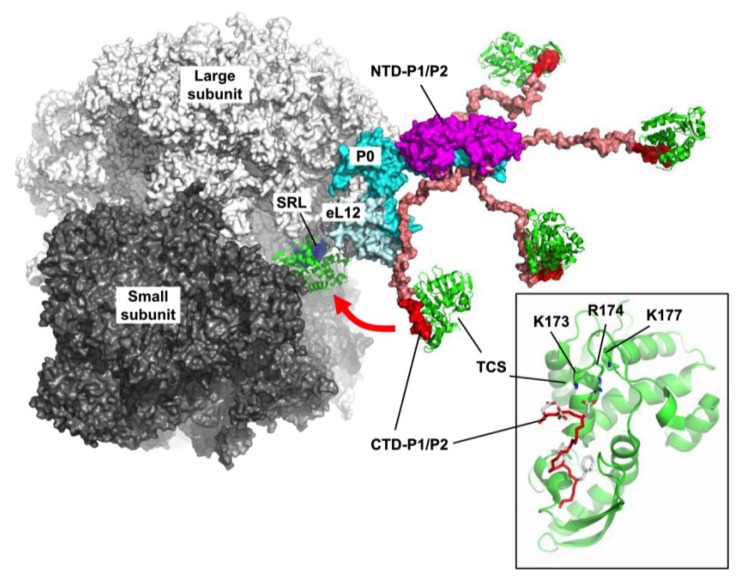
Structural insights into how eukaryotic stalk recruits trichosanthin (TCS) to the α-sarcin/ricin loop (SRL). The structural model of eukaryotic stalk complex created as described [27] was docked to the crystal structure of yeast ribosome [22]. P0 (cyan) binds two copies of P1/P2 heterodimers via their *N*-terminal domains (NTD) (magenta). The *C*-terminal domain (CTD) of P1/P2 (red) is connected to the NTD via a flexible linker (salmon). The consensus sequence (SDDDMGFGLFD) at the CTD forms a complex with trichosanthin (green), in which the K173, R174 and K177 form favorable charge-charge interactions with the DDD motif of P1/P2 (inset). The normal function of the hydra-like structure of eukaryotic stalk, which can extend up to 125 Å from the stalk base, is to recruit elongation factors to GTPase association center of ribosomes. It is postulated that TCS gains access to the SRL by hijacking this elongation-factor-recruiting machinery of eukaryotic ribosomes.

Despite recent advances in the structural studies of ribosomes [33,34], the CTD of ribosomal stalks are not defined due to their intrinsic flexibility. Molecular modelling was used to generate structural models of ribosomal stalks by fitting the structures of individual stalk protein complexes to the structures of bacterial [11], archaeal [17] and eukaryotic [27] ribosomes. For example, we have recently determined the solution structure of full-length human P1/P2 heterodimer by NMR spectroscopy [27]. While the *N*-terminal dimerization domain of P1/P2 is well structured, the *C*-terminal tails of P1/P2 are disordered and can extend up to 125 Å away from the dimerization domain. NMR relaxation measurement showed that the *C*-terminal tails are flexible with significantly faster effective correlation time for the reorientation of the backbone amide group [27]. A common structural insight derived from these structural models is that multiple copies of the CTD of stalk proteins (P1/P2 or L12) are connected via a flexible linker region to the NTD, which binds to the spine-helix of L10 or P0. Presumably, the hydra-like structures of ribosomal stalk and the long flexible linkers allow the CTD of stalk proteins to reach out and fetch elongation factors to the GTPase association center of ribosomes, where GTP hydrolysis is stimulated to drive protein synthesis (Figure 2) [11,17].

## 4. Trichosanthin Hijacks the Eukaryotic Stalk Proteins by Binding to Their *C*-Terminal Consensus Sequences

Trichosanthin (TCS) is a type I RIP derived from the root tuber of *Trichosanthes kirilowii*, a traditional Chinese herbal medicine. Like ricin, TCS can only inactivate eukaryotic ribosomes but not bacterial ribosomes. The pharmacological properties of TCS have been reviewed by Shaw and co-workers [35]. Here, we review how we used TCS as a model to study how RIP hijacks the normal function of eukaryotic stalk and gain access to the SRL of eukaryotic ribosomes.

The initial evidence that TCS interacts with ribosomal stalk proteins came from a yeast-two-hybrid studies by Chan and co-workers [36]. An inactive mutant (E160A/E189A) of TCS was used to screen cDNA libraries derived from human placenta cells, and identified P0 and P1 as the interacting partners of TCS. Later, chemical-shift perturbation and systematic truncation studies have shown that TCS interacts with the highly conserved sequence, SDDDMGFGLFD, located at the *C*-terminus of human P2 [37]. Interestingly, this conserved sequence is also present in P0 and P1. Site-directed mutagenesis studies demonstrated that three basic residues, K173, R174 and K177 at the *C*-terminal domain of TCS and the DDD motif of P2 are important in the interaction between TCS and P2. Crystal structure of TCS in complex with the consensus sequence SDDDMGFGLFD revealed that the DDD motif forms favorable electrostatic interactions with the K173, R174 and K177 residues of TCS (Figure 2, inset) [38].

Moreover, the LF motif of P2 is docked into a hydrophobic pocket at the *C*-terminal domain of TCS. Mutations that broke the interactions between TCS and P2 also abolished the *N*-glycosidase activity of TCS and significantly reduced the ability of TCS to inactivate ribosome *in vitro* [37,38]. These observations strongly suggest that interaction between TCS and eukaryotic stalk proteins are important in the ribosome-inactivating properties of TCS.

Using the hybrid ribosome methodology, we have recently demonstrated that the ribosomal stalk plays a crucial role in the eukaryote-specific action of TCS [27]. Bacterial ribosomes are insensitive to TCS. Replacing the bacterial stalk proteins (L11 and L10/L12 complex) with eukaryotic counterparts (eL12 and P0 (P1/P2)_2_ complex) made the hybrid ribosome sensitive to the *N*-glycosidase activity of TCS. On the other hand, shortening or truncation of the flexible linker resulted in ribosomes insensitive to the action of TCS [27]. These results suggest that both the CTD and flexible linker of eukaryotic stalk proteins are responsible for recruiting these RIPs to the SRL where the toxin can carry out its *N*-glycosidase activity. 

Uchiumi and co-workers have recently determined the crystal structure of archaeal EF1α (aEF1α) in complex with the CTD of archaeal P1 (aP1) [39]. The CTD of aP1 forms a helix that docks to a cleft between domain I and III of aEF1α. In contrast, the GFGL motif of human P1/P2 CTD adopts a β-turn conformation when in complex with TCS [38]. The structure of the CTD of eukaryotic P1/P2 in complex with elongation factors is currently not known. However, there are notable differences in the sequences of eukaryotic P1/P2 (SDDDMGFGLFD) and aP1 (EALAGLSALFG): (1) the DDD motif, responsible for binding TCS, in P1/P2 is replaced by hydrophobic residues in aP1; (2) The GFGL motif, which is highly conserved among eukaryotic stalk proteins, is not found in aP1. These sequence differences suggest that there could be real structural differences between the CTD of eukaryotic P1/P2 and aP1. Despite the observed differences, one common trend was observed—The LF motif, which is conserved in eukaryotic and archaeal stalk proteins, was shown to be responsible for binding both archaeal and eukaryotic translation factors [10,39] as well as for binding TCS [38] via hydrophobic interactions. It is likely that TCS takes advantage of this conserved sequence motif in order to get access to the eukaryotic ribosomes.

## 5. Interaction with Stalk Proteins is also Required for the Toxic Action of Other RIPs

Apart from TCS, other RIPs are also found to mediate their toxicity via binding to the stalk proteins. By surface plasmon resonance, Tumer’s group showed that the interaction between ribosomes and ricin-A-chain (RTA) was greatly abolished in the absence of P1 and P2, and such interaction is important in the *in vivo* toxicity of RTA in yeast and in human cells [40,41]. Using surface plasmon resonance, they showed that the association rate between RTA and wild-type ribosomes from yeast is very fast (on the order of 10^7^ M^−1^·s^−1^). However, for mutant ribosomes lacking P1/P2, the association became slower by ~100 folds. Their results support a two-step binding model—the fast step is dependent on stalk proteins P1/P2, while the slower step is not [42].

Like TCS and ricin, Shiga toxin 1 A-chain (Stx-1A) was also found to interact with P0, P1 and P2 by pull-down experiment [43]. The binding site of stalk proteins with RTA and Stx-1Awas mapped to the conserved *C*-terminal 11 residues of stalk proteins by *in vitro* pull-down assay [43]. In the crystal structure of TCS in complex with the conserved *C*-terminal residues of P1/P2, the DDD motif of the stalk proteins forms favorable electrostatic interaction with the basic residues of TCS. It has been shown that high salt concentration can abolish the interaction between RTA and yeast ribosomes, suggesting that the interaction is electrostatic in nature [42]. Taken together, it is likely a general theme that RIPs take advantage of the highly conserved *C*-terminal acidic residues of stalk proteins, which facilitate their recruitment to the ribosomes. Indeed, this suggestion has been supported by mutagenesis studies in TCS, Stx-1A, and maize RIP [37,44,45].

## 6. Conclusions

The analogy between the recruitment of elongation factors and TCS to the ribosomes is striking. In both cases, the target binding site within the ribosome is the SRL and the CTD of eukaryotic stalk proteins are involved in specific recognition. The similarity prompted us to postulate that some eukaryote-specific RIPs like TCS and ricin may have evolved to bind the CTD of eukaryotic stalk proteins, hereby hijacking the elongation-factor-recruiting machinery of eukaryotic stalk for targeting to the SRL of ribosomes (Figure 2).

Although recent structural and biochemical studies have suggested the crucial role of eukaryotic stalk in recruiting TCS to the ribosomes, other ribosomal proteins may also play a role in facilitating the action of RIPs. For example, Stx-1A can inhibit protein synthesis of both eukaryotic and bacteria ribosomes with similar potency [46], indicating that stalk proteins may not be the only target for Stx-1A. Moreover, Shiga toxin 2 A-chain (Stx-2A), which shares only 55% sequence identity to Stx-1A, is less dependent on the stalk proteins for activity [47]. For example, the depurination activity of Stx-1A on ribosome was greatly abolished if P1/P2 binding sites on P0 were truncated while the depurination activity of Stx-2A was not much affected by this truncation [47]. On the other hand, in the presence of P1/P2 binding sites, addition of extra P1/P2 increase the depurination activity of Stx-1A more than that of Stx-2A [47]. Tumer and co-workers have shown that ribosomal protein L3 is required for ribosomal inactivating activity of pokeweed antiviral protein [48]. Interestingly, L3 is conserved in bacterial and eukaryotic ribosomes, which may justify the observation that pokeweed antiviral protein can inactivate both ribosomes. On the other hand, native eukaryotic ribosomes from *Artemia* is much more sensitive to TCS than the hybrid ribosome, which contains the eukaryotic stalk proteins on the bacterial 50S core ribosomes, suggesting other ribosomal proteins should play a role in facilitating the action of RIPs [27].
